# Advances in artificial intelligence applications in the field of lung cancer

**DOI:** 10.3389/fonc.2024.1449068

**Published:** 2024-09-06

**Authors:** Di Yang, Yafei Miao, Changjiang Liu, Nan Zhang, Duo Zhang, Qiang Guo, Shuo Gao, Linqian Li, Jianing Wang, Si Liang, Peng Li, Xuan Bai, Ke Zhang

**Affiliations:** ^1^ Clinical Medical College of Hebei University, Affiliated Hospital of Hebei University, Baoding, China; ^2^ Thoracic Surgery Department, Affiliated Hospital of Hebei University, Baoding, China; ^3^ Thoracic Surgery Department, Fourth Hospital of Hebei Medical University, Shijiazhuang, China; ^4^ Basic Research Key Laboratory of General Surgery for Digital Medicine, Affiliated Hospital of Hebei University, Baoding, China; ^5^ Information center, Affiliated Hospital of Hebei University, Baoding, China; ^6^ Institute of Life Science and Green Development, Hebei University, Baoding, China; ^7^ 3D Image and 3D Printing Center, Affiliated Hospital of Hebei University, Baoding, China; ^8^ Department of Radiology, Affiliated Hospital of Hebei University, Baoding, China

**Keywords:** artificial intelligence, lung cancer, machine learning, deep learning, convolutional neural network

## Abstract

Lung cancer remains a leading cause of cancer-related deaths globally, with its incidence steadily rising each year, representing a significant threat to human health. Early detection, diagnosis, and timely treatment play a crucial role in improving survival rates and reducing mortality. In recent years, significant and rapid advancements in artificial intelligence (AI) technology have found successful applications in various clinical areas, especially in the diagnosis and treatment of lung cancer. AI not only improves the efficiency and accuracy of physician diagnosis but also aids in patient treatment and management. This comprehensive review presents an overview of fundamental AI-related algorithms and highlights their clinical applications in lung nodule detection, lung cancer pathology classification, gene mutation prediction, treatment strategies, and prognosis. Additionally, the rapidly advancing field of AI-based three-dimensional (3D) reconstruction in lung cancer surgical resection is discussed. Lastly, the limitations of AI and future prospects are addressed.

## 1 Introduction

Lung cancer is a prevalent malignancy with high incidence and mortality rates worldwide. According to the latest statistics from GLOBOCAN (1), lung cancer ranks second in terms of incidence and first in terms of mortality among all malignant tumors. In 2020, there were over 2.2 million new cases of lung cancer globally, with approximately 1.8 million deaths. This disease poses a significant threat to human health and life. However, early-stage lung cancer is often challenging to detect based on symptoms, leading to the majority of patients being diagnosed at an advanced stage or with distant metastases, resulting in a five-year survival rate of only 15-16% (2). Consequently, the limitations in treatment options and prognosis assessment present challenges for clinicians. Early diagnosis and appropriate treatment are crucial factors in reducing lung cancer mortality.

With the continuous development of imaging and computer technology, X-ray computed tomography (CT) has been widely used in clinical practice, and population-based lung cancer screening has demonstrated high cost-effectiveness and significant value for early prediction in the community. Data from the National Lung Screening Trial (NLST) have shown that regular screening of high-risk individuals for lung cancer using low-dose CT imaging can detect more early-stage cancers and reduce lung cancer-related mortality (3).

In recent years, artificial intelligence (AI) technology has found extensive applications across various industries, especially in the healthcare sector (4). The combination of “AI + healthcare” has become a prominent trend, encompassing areas such as assisted diagnosis, risk prediction, treatment selection, and outcome evaluation (5, 6). By utilizing AI technology to analyze extensive medical data and train specialized AI models, it is possible to significantly enhance healthcare professionals’ diagnostic efficiency and accuracy. Moreover, AI can identify patterns and features that may be overlooked in manual diagnosis, thereby providing clinicians with more comprehensive and detailed diagnostic and treatment recommendations. This review aims to outline the advancements in the application of artificial intelligence in the field of lung cancer.

## 2 Overview of AI

AI is a branch of computer science that explores and develops theoretical methods for simulating, extending, and enhancing human intelligence, with the aim of automating tasks that are typically performed by humans (7). Since the 1950s, AI has undergone three major stages: symbolism (1950s to 1980s), the flourishing development of ML (1990s), and the significant success of DL in the early 21st century. Over time and with technological advancements, symbolic AI has gradually taken a backseat, making room for ML and DL as the core subfields in the field of AI. **Table 1** lists the key terms associated with AI.

**Table 1:** Key terms and major functions in artificial intelligence.

Keywords	Definition
AI	A field of computer science that researches and develops systems capable of simulating and implementing human intelligence.
ML	Enabling computers to learn from data and automatically recognize and grasp patterns and regularities within the data, in order to make predictions and decisions.
SL	Supervised learning is a machine learning method in which a model learns the relationship between inputs and outputs from labeled training data.
UL	Unsupervised learning is a machine learning paradigm where the model learns the structure, patterns, and relationships within unlabeled data without the need for labels or predefined outputs.
RL	Reinforcement learning is a method where an agent interacts with an environment, learning to optimize its behavior through trial and error.
DL	A technique that employs multi-layered structures called deep neural networks to automatically extract and learn features from data, transmitting and processing information through connections between layers to produce the final output.
CNN	A type of deep learning model designed specifically for processing data with grid-like structures, such as images and videos, using components like convolutional layers, pooling layers, and fully connected layers to extract and learn features from the data.
RNN	A type of neural network used for processing sequential data, such as speech and text. It features recurrent connections that can remember previous information and make predictions based on the current input.

ML is one of the core technologies in the field of Artificial Intelligence, aiming to enable computers to learn from data, automatically identify and grasp patterns and regularities within the data, thereby achieving the capability of prediction and decision-making. ML is generally categorized into three types: supervised learning (SL), unsupervised learning (UL), and reinforcement learning (RL). SL algorithms use classified training data to create a prediction function that can generalize to classify unseen data correctly. UL differs in that no target variable exists. All variables are treated as inputs, and therefore unsupervised learning is used to find patterns in the data (8). RL is the process in which computers learn to complete tasks by learning from the outcomes of successes and failures. Common ML algorithms include support vector machines, random forests, decision trees, logistic regression, k-nearest neighbors, Bayesian networks, and clustering algorithms etc.

**Figure 1:**
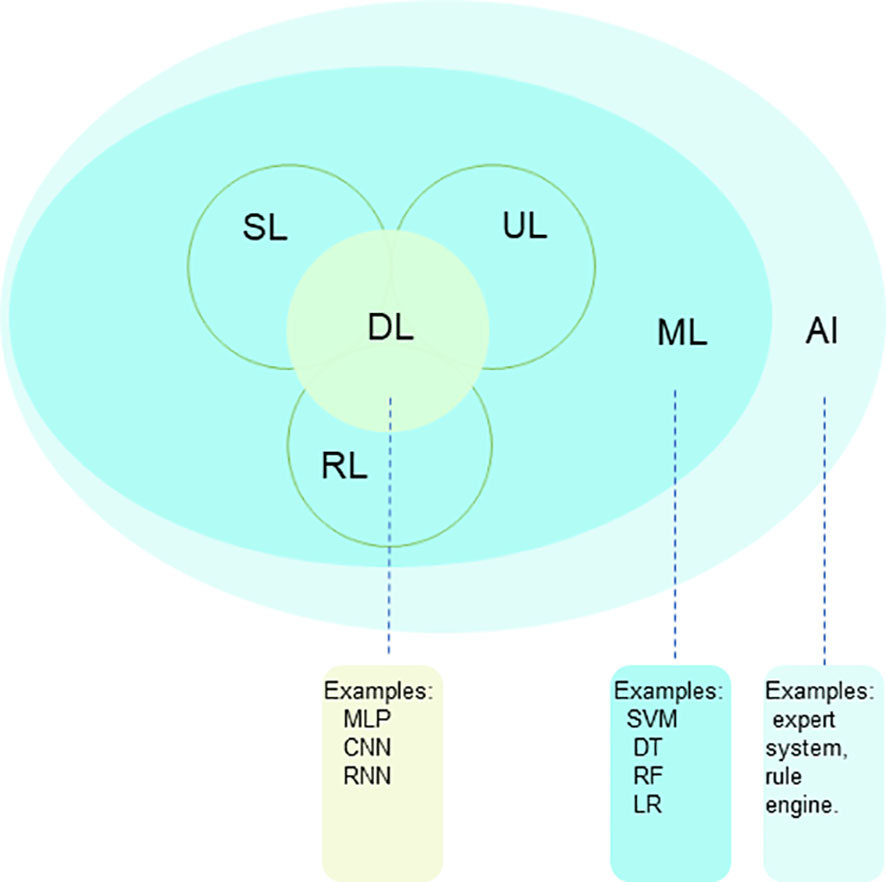
The relationship between AI, ML and DL, which are methodologies and technologies that enable computers to learn, analyze patterns, understand languages and images, and make predictions. AI, artificial intelligence; ML, machine learning; DL, deep learning; SL, supervised learning; UL, unsupervised learning; RL, reinforcement learning.

DL is a subfield of ML (**Figure 1**) that uses multi-layer artificial neural networks (ANN) to recognize patterns in data. Unlike traditional ML algorithms, DL algorithms can automatically learn features from raw data for pattern recognition. They learn abstract features through multiple layers of stacking, making models more powerful. The structure of DL models allows them to automatically learn and extract features from data, performing well on complex tasks and large datasets. During training, gradient descent and backpropagation are commonly used to adjust the model’s parameters, improving performance by reducing the difference between predicted and actual results. Common DL models include Convolutional Neural Networks (CNNs) and Recurrent Neural Networks (RNNs). CNNs are mainly used for image-related tasks, while RNNs are good at processing text and speech. These models are essential in DL and have many variations and combinations. They provide powerful tools for solving problems in areas like computer vision and natural language processing.

AI and radiomics are two closely related but distinct fields. AI aims to develop systems that simulate human intelligence and is widely applied in medical diagnostic support, medical imaging analysis, and more. Radiomics, on the other hand, focuses on extracting quantitative features from medical images and combining them with clinical data to predict disease and assess prognosis. AI is a broader field, while radiomics specifically concentrates on medical image analysis.

## 3 Application of AI in lung cancer diagnosis

### 3.1 Application of AI in lung nodule detection

Early-stage lung cancer is closely related to lung nodules. Lung nodules are typically detected through chest CT scans. On CT scans, a nodule appears as a rounded or irregular opacity, well or poorly defined, measuring up to 3 cm in diameter (9). Although most lung nodules are benign, some may develop into lung cancer. The National Lung Screening Trial has shown that lung cancer screening can reduce mortality rates among high-risk populations (3). Therefore, the increased use of lung cancer screening will inevitably lead to the discovery of many pulmonary nodules whose malignancy is uncertain. For incidentally detected nodules, the most commonly used guidelines are those of the Fleischner Society (10) and the British Thoracic Society (BTS) (11). The Fleischner Society guidelines are based on the mean diameter and type of the nodule, while the BTS guidelines recommend volumetric measurement of nodules rather than 2D measurements.

However, identifying lung nodules is not always easy, and radiologists’ sensitivity in detecting these nodules varies significantly, which can be influenced by various characteristics such as size, shape, location, density, and their relationship with adjacent structures (12). A large number of manual image readings can lead to missed diagnoses. Nowadays, there is growing interest in using AI to detect lung nodules. In lung cancer screening, AI can be applied not only for automatic detection, but also for patient selection and the reconstruction of low-dose CT scans (13). Kim et al. (14) have shown that an AI-based tool can improve the performance of radiologists and pulmonologists when estimating malignancy risk for indeterminate pulmonary nodules on chest CT scans. Moreover, application to predict benignity/malignity of the nodules are also available (15). However, AI-based tools require further research to determine their diagnostic benefits for clinicians in assessing indeterminate pulmonary nodules on chest CT scans (16).

**Figure 2:**
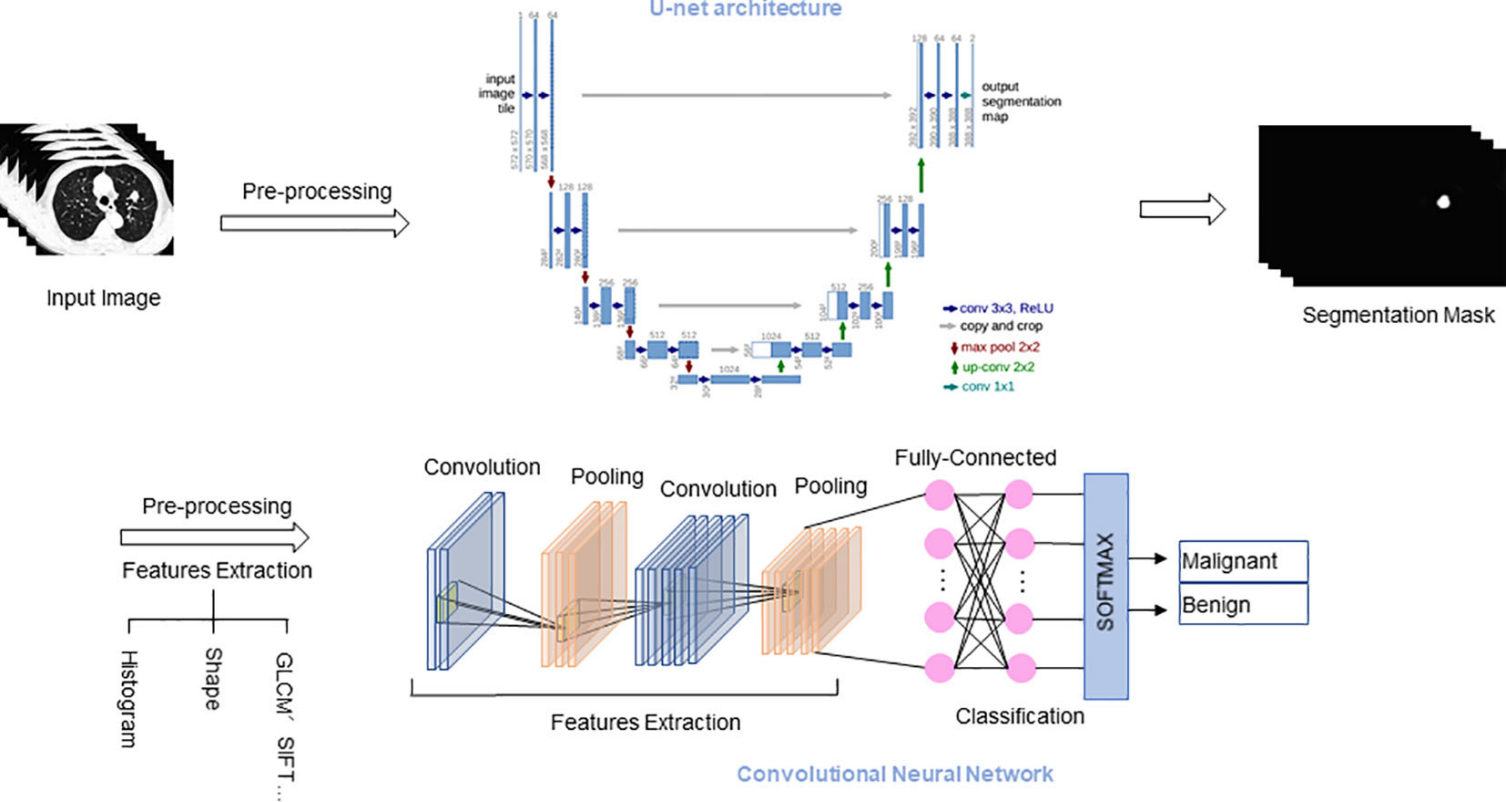
Segmentation and classification of lung nodules. U-Net architecture for segmentation tasks, and CNN architecture for classification tasks. GLCM, Gray-level co-occurrence matrix; SIFT, Scale-invariant feature transform.

Lung nodule CT images are multi-dimensional. 3D CNN can better utilize the 3D spatial information of the input, enabling tasks such as detection, segmentation, and classification of 3D objects, thereby improving detection accuracy. Khosravan et al. (17) proposed a novel DL-based method for lung nodule detection called S4ND. The whole detection pipeline is designed as a single 3D CNN with dense connections, trained in an end-to-end manner. This method uses a single feedforward pass of a single network to detect lung nodules without further processing, achieving a sensitivity of 95.2%. Subsequently, following detection, the focus shifts to precisely delineating or segmenting lung nodules from the adjacent pulmonary parenchyma. Segmentation entails separating nodules from other structures in the image, thus facilitating their comprehensive analysis and precise measurement of relevant characteristics. Manual segmentation is time-consuming and highly variable between observers. AI-based methods, including U-Net and other DL models, can replace this process to objectively quantify lung nodules and cancer, and extract radiomic features for tissue characterization (18). Ronneberg et al. (19) developed the U-Net, which is a CNN architecture, for biomedical segmentation tasks. U-Net can be used to perform fine pixel-level segmentation of detected lung nodules, accurately outlining the spatial extent of nodules, laying the foundation for subsequent quantitative analysis and growth assessment. Bhattacharyya et al. (20) employed a weighted bidirectional feature network to construct an improved U-NET architecture (DB-NET), which demonstrates excellent performance in lung nodule segmentation. Through this approach, they achieved better performance in segmenting ground-glass nodules, cavitary nodules, small nodules. Lastly, lung nodules are classified into distinct categories, including benign or malignant, solid or subsolid, and specific subtypes, based on their respective features. In order to improve the accuracy of classifying benign and malignant lung nodules, Guo et al. (21) proposed a 3D segmentation attention network integrating asymmetric convolution (SAACNet) classification model combined with a gradient boosting machine (GBM). The proposed nodule classification performance was evaluated on the LUNA16 dataset, which contains 888 CT scan images, achieving a classification accuracy of 95.18%, and the area under the curve (AUC) is 0.977. **Figure 2** depicts the segmentation and classification of lung nodules.

**Table 2** presents a summary of applications of AI in lung nodules. These AI methods each have their own advantages and disadvantages. k-medoids clustering is robust to noise and outliers but is computationally intensive and sensitive to initial selection. CNNs perform well in image processing but require large amounts of labeled data and high computational resources. 3D CNNs can handle three-dimensional medical images, capturing more spatial information, but have higher computational costs. DenseNet improves information flow through dense connections, but the complexity of the model increases. 3D U-NET is suitable for medical image segmentation but has longer training times.

**Table 2:** Applications of AI in lung nodules.

Ref.	Year	Purpose	Dataset	Methods	Results
Uthoff et al. (22)	2019	Lung nodule classification.	Training:74 malignant and 289 benign, Validation:50 malignant and 50 benign.	k-medoids clustering and information theory.	AUC = 0.965, Sensitivity: 100% Specificity: 96%
Nasrullah et al. (23)	2019	Lung nodule detection and classification.	LIDC-IDRI LUNA16.	3D CMixNet	Sensitivity:94% Specificity:91%
Gong et al. (24)	2020	To classify GGN as IA or non-IA.	828 GGNs.	3D SE-ResNet	AUC= 0.92 ± 0.03.
Naqi et al. (25)	2020	Lung nodule detection and classification.	LIDC-IDRI 1018 Cases.	3D CNN.	Sensitivity:95.6%
Zhang et al. (26)	2021	Lung nodule classification.	888 CT scans.	DenseNet.	Accuracy:92.4%
Zheng et al. (27)	2021	Lung nodule detection.	1186 nodules.	U-net++	Sensitivity: 94.2%
Hu et al. (28)	2021	To classify between benign and malignant GGNs.	513 GGNs.	3D U-NET, deep neural network.	Accuracy:75.6%
Ahmed et al. (29)	2022	Pulmonary nodules classification and Detection.	1018 cases of lung cancer.	Classification:VGG-16, Mobilenet, Resnet50. Detection: YOLOv3, Faster-RCNN, SSD.	Classification: average accuracy 92%~95% Detection: average accuracy 93%~94%
Luo et al. (30)	2022	Lung nodule detection.	888 CT scans.	SCPM-Net.	Average sensitivity: 89.2%
Li et al. (31)	2022	Pulmonary nodules segmentation.	1487 CT images.	REMU−Net.	Accuracy:99.02%
Saied et al. (32)	2023	Pulmonary nodules classification.	1007 nodules.	10ML and 6DL.	ML: SVM (81.9 ± 1.6%) DL: DenseNet-121 (90.39%)
Tang et al. (33)	2023	Pulmonary nodules segmentation and feature extraction.	1,186 nodules.	SVM, RF, SVR.	Overall accuracy:75%

AUC, Area under a ROC curve; 3D CMixNet, three-dimensional customized mixed link network; SE-ResNet, squeeze-and-excitation network and residual network; CNN, Convolutional Neural Network; DenseNet, Dense Convolutional Network; DL, Deep Learning; Faster RCNN, Faster Region-based Convolutional Neural Network; GGNs, ground-glass nodules; IA, invasive adenocarcinoma; LIDC-IDRI, Lung Image Database Consortium and Image Database Resource Initiative; LUNA16, Lung Nodule Analysis 16; ML, Machine Learning; ResNet, Residual network; ResNeSt, A variant of ResNet; REMU−Net, ResNeSt-SAM enhancement module multi-scale skip connection U−Net; RF, Random forest; SCPM-Net, 3D sphere center-points matching detection network; SSD, Single Shot Detector; SVM, Support vector machine; SVR, Support vector regression; VGG, Visual Geometry Group; YOLOv3, You Only Look Once version 3.

### 3.2 Application of AI in lung cancer pathology diagnosis

Lung cancer is mainly classified into non-small cell lung cancer (NSCLC) and small cell lung cancer (SCLC), with NSCLC accounting for approximately 80%-85% of all lung cancers (34). NSCLCs are classified according to histopathological characteristics. Lung adenocarcinoma (LUAD) and lung squamous cell carcinoma (LUSC) are the most common subtypes of NSCLC. Histopathologic confirmation remains the gold standard in clinical workflow for diagnosis (35), and due to significant differences between different subtypes, clinical treatments also vary greatly. Therefore, accurate classification of lung cancer subtypes is crucial. Pathologists examine tissue samples from lung biopsies and resections to detect and classify cells, identify tumor morphology and subtypes, and assess features that predict treatment response and prognosis (36). Manual slide reading is time-consuming and prone to fatigue, while the development of AI-based tools can assist pathologists and thoracic surgeons in improving clinical workflows and patient management.

It has been demonstrated that AI can accurately classify lung cancer subtypes and predict the prognosis of NSCLC patients. Yu et al. (37)used histopathology whole-slide images of LUAD and LUSC patients from The Cancer Genome Atlas (TCGA) to extract a large number of image features. They employed regularized ML methods to select the most important features to differentiate short-term survivors from long-term survivors. Their study results indicate that automatically extracted image features can predict the prognosis of lung cancer patients, thus contributing to precision oncology. This method can also be applied to histopathology images of other organs. Similarly, Coudray et al. (38)trained a deep CNN (Inception v3) with 1,634 randomly selected histopathological whole-slide images from the TCGA database to classify them as LUAD, LUSC, or normal lung tissue based on their morphological features. The results were consistent with the analysis of pathologists, with an average AUC of 0.97.

AI tools have also been applied to cytopathological samples obtained through fine-needle aspiration, assisting in the diagnosis of lung cancer and differentiation from other diseases by analyzing various samples such as bronchial secretions, sputum, bronchoalveolar lavage fluid, and needle aspiration. Compared to tissue biopsies, this examination is less invasive and is increasingly being used for lung cancer assessment and staging (39). Gonzalez et al. (40)studied cytological and biopsy specimens from 40 patients and trained a DL algorithm based on CNN to differentiate SCLC from large-cell neuroendocrine carcinoma based on morphological features. Although the dataset used was small, the DL models employed in the study accurately identified the majority of cases of both tumor types.

The spatial distributions of different types of cells could reveal a cancer cell’s growth pattern, its relationships with the tumor microenvironment and the immune response of the body. Wang et al. (41)developed a software tool for LUAD digital pathological image analysis aided by a CNN, ConvPath, which includes nuclei segmentation, CNN-based tumor cell, stromal cell, and lymphocyte classification, and extraction of tumor microenvironment-related features for lung cancer pathology images. Moreover, it has the capability to convert the pathology image into a “spatial map” of tumor cells, stromal cells and lymphocytes. This could greatly facilitate and empower comprehensive analysis of the spatial organization of cells, as well as their roles in tumor progression and metastasis. The overall classification accuracy was 92.9% and 90.1% in training and independent testing datasets, respectively. They also developed and validated a prognostic model, enabling personalized treatment planning for individual patients using readily available tissue images, which proves to be highly beneficial for pathologists and clinical practitioners.

### 3.3 Application of AI in predicting gene mutations in lung cancer

In recent years, researchers have utilized ML and DL techniques to analyze large-scale lung cancer genomic data, exploring gene variations associated with the occurrence and development of lung cancer, and predicting genes that may serve as driver mutations. LUAD is driven by a series of accumulated genetic changes known as driver mutations, such as mutations in epidermal growth factor receptor (EGFR), kirsten rat sarcoma viral oncogene homolog (KRAS), and anaplastic lymphoma kinase (ALK) fusion (42–44), which are potential therapeutic targets. Coudray et al. (38)downloaded gene mutation data from TCGA matched with patient samples and trained a CNN model to predict the 10 most common mutated genes in LUAD. The results showed that six of them (STK11, EGFR, FAT1, SETBP1, KRAS, TP53) could be predicted using pathological images, with AUC ranging from 0.733 to 0.856. In another study, Wang et al. (45)developed an innovative AI system known as the Fully Automated Artificial Intelligence System (FAIS) to predict EGFR gene mutations in lung cancer, as well as to assess the progression-free survival (PFS) of patients receiving EGFR-targeted therapy. By utilizing non-invasive CT images, the FAIS achieved promising predictive performance with AUC values ranging from 0.748 to 0.813 for EGFR gene mutations and demonstrated statistically significant associations with the PFS of patients (log-rank p<0.05). The FAIS efficiently achieved these predictions without requiring manual annotation, highlighting its potential in advancing lung cancer diagnostics and personalized therapy.

In addition to the common EGFR and KRAS gene mutations, ALK fusions are also observed in NSCLC. Song et al. (46)conducted a study in which they trained and validated a DL model using CT images and clinical pathological information to predicting the ALK fusion status in 937 NSCLC patients, achieving an AUC of 0.8046. Furthermore, the study predicted the prognosis of 91 patients undergoing ALK-TKI drug therapy, revealing that ALK-positive patients experienced longer PFS (16.8 vs. 7.5 months, *P* = 0.010).

Overall, the application of AI in predicting gene mutations in lung cancer demonstrates significant potential. Utilizing AI models can not only rapidly detect and identify gene mutations to formulate personalized treatment plans but also predict patients’ treatment responses and prognosis. These capabilities not only improve the effectiveness of diagnosis and treatment but also reduce the waste of medical resources. In the future, AI models are expected to discover new therapeutic targets and further enhance treatment precision by integrating multiple data sources.

## 4 Application of AI in lung cancer treatment

### 4.1 Application of AI-based 3D reconstruction in lung cancer

3D reconstruction technology, as part of AI-assisted surgery, has been increasingly used in clinical practice. By utilizing computer software to reconstruct 3D images from various imaging modalities such as CT, magnetic resonance imaging (MRI) and positron emission tomography-computed tomography (PET-CT), clearer and more intuitive 3D models of thoracic lesions and surrounding structures can be obtained, aiding in preoperative evaluations. This technology boosts the confidence of medical professionals and provides valuable assistance in formulating surgical plans and conducting surgical procedures. It has been reported that 20-30% of patients have variations in pulmonary vasculature (47). Therefore, the identification of anatomical variations is essential during preoperative planning or intraoperative navigation in lung cancer surgery. In a retrospective cohort study, thoracic surgeons achieved an accuracy rate of 85% in identifying anatomical variations using AI-assisted CT, with a median time of 2 (1–3) minutes (48). AI-driven reconstruction enables surgeons to achieve a high level of accuracy in identifying anatomical patterns within a short period, which has practical value in surgical planning for segmentectomy. CT-based pulmonary broncho-vascular 3D reconstruction facilitates precise anatomical pulmonary segmentectomy, making complex lung segmentectomy via a thoracoscopic approach safer and more convenient. This is especially advantageous for patients with deep nodules and vascular anatomical variations, where the benefits of 3D reconstruction are particularly evident.

Lung 3D reconstruction models can be created using semi-automatic tools such as Mimics, OsiriX, and 3DSlicer, which simulate anatomical structures, define lung segment divisions, and determine lesion locations. Intraoperative navigation using accurate 3D reconstruction models significantly reduces surgical time and improves surgical success rates (49). However, the high level of expertise required and the substantial time consumption may limit the widespread clinical application of these systems. AI technology can automatically learn from raw data and rapidly generate 3D models, enhancing clinical efficiency. Currently, AI-based 3D reconstruction software has been developed. Li et al. (50)constructed a fully automated 3D reconstruction system based on 3D CNN to assist thoracic surgery and to determine its accuracy, efficiency, and safety for clinical use. The AI system resulted in a significant reduction in operation time by 24.5 min for lobectomy (*P* < 0.001) and 20 min for segmentectomy (*P* = 0.007). Compared to manual reconstruction software (Mimics), the AI system reduced the model reconstruction time by 14.2 min (*P* < 0.001), and it also outperformed Mimics in model quality scores (*P* < 0.001).

**Figure 3:**
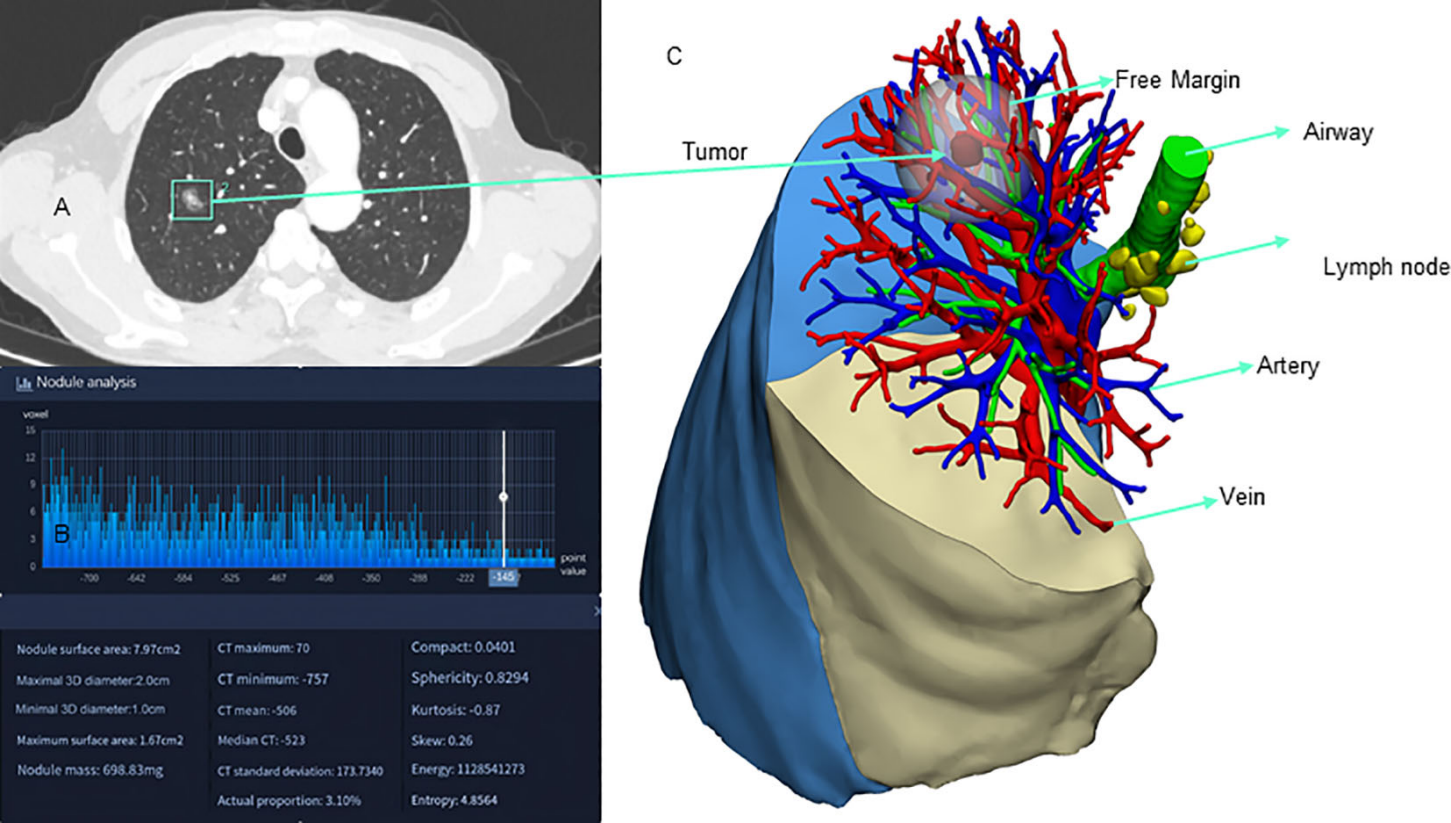
Application of artificial intelligence 3D reconstruction in lung cancer. **(A)** AI-assisted nodule localization; **(B)** AI-assisted nodule analysis; **(C)** AI-assisted 3D reconstruction.

Currently, AI-based 3D reconstruction systems mostly focus on the automatic reconstruction of pulmonary vessels and bronchi (48, 50, 51). There are no reported studies on automatic 3D reconstruction systems for tissue structure changes after neoadjuvant therapy, which could be one of the future development directions for AI in the field of lung cancer 3D reconstruction. In the future, with the continuous development of AI technology, various devices such as AI-guided robotic surgery systems, AI-assisted lung cancer biopsy and treatment robots, and automated lung cancer surgery robots may become a reality in this field. **Figure 3** illustrates the application of AI in 3D reconstruction for lung cancer.

### 4.2 AI-assisted preoperative assessment

Surgical resection, radiation therapy, and chemotherapy are common treatment approaches for lung cancer. In recent years, there have been significant advancements in molecular biology and genomics, leading to the widespread use of targeted therapy, immunotherapy, neoadjuvant therapy, and other treatment modalities in clinical practice. These advancements have resulted in improved patient prognosis to some extent. The selection of lung cancer treatment strategies generally relies on histopathological classification, immunohistochemical markers, and Tumor Node Metastasis (TNM) staging. However, despite having similar clinical characteristics, patients can exhibit significant individual differences in their response to the same treatment strategy. Therefore, early assessment and prediction of treatment efficacy for various treatment strategies are particularly crucial. AI-assisted preoperative assessment facilitates more accurate treatment decisions for doctors and provides patients with more personalized treatment plans.

The preoperative diagnosis of invasiveness is still difficult in the clinical setting since the pathological invasiveness of lung cancer is evaluable only after scrutinizing the pathological specimen. AI models can be utilized to achieve preoperative diagnosis of invasiveness. Onozato et al. (52)enrolled 873 patients who underwent lobectomy or segmentectomy for primary lung cancer and extracted the radiomic features from preoperative PET and CT images. They compared seven ML models and an ensemble model (ENS) combining PET and CT features. The results showed that all models achieved an AUC of ≥0.880 in predicting tumor invasiveness in the training set. In the test set, ENS showed the highest mean AUC of 0.880 and accuracy of 0.804.

In other studies, AI models demonstrated radiologist-level performance in predicting visceral pleural invasion (53) and identifying early-stage LUAD suitable for sublobar resection (54). Lv et al. (55)developed a deep learning model that achieved comparable performance to intraoperative frozen section analysis in determining tumor invasiveness. The proposed method may contribute to clinical decisions related to the extent of surgical resection. Due to the lack of risk stratification models for invasive adenocarcinoma, Zhou et al. (56)proposed an Ensemble Multi-View 3D Convolutional Neural Network (EMV-3D-CNN) model to study the risk stratification of lung adenocarcinoma. Their model outperformed senior physicians in risk stratification of invasive adenocarcinoma, achieving an accuracy of 77.6%. This provides detailed predictive histological information for the surgical treatment of pulmonary nodules.

### 4.3 AI-predicted immunotherapy efficacy

Immune checkpoint inhibitors (ICIs) targeting programmed death-1 (PD-1) or programmed death-ligand 1 (PD-L1) have become the mainstay of therapy for NSCLC patients without targeted treatment options, although only 20-40% of patients benefit from these new therapies (57). Immunotherapy has shown the potential to improve the prognosis of lung cancer patients, significantly prolonging PFS and overall survival (OS) in advanced NSCLC patients. Despite these promising results, the use of ICI remains constrained by its high costs and toxicity. Additionally, its clinical efficacy is limited to a small subset of patients, primarily assessed by monitoring the PD-L1 expression levels in tumor cells. The KEYNOTE-042 trial demonstrated a strong correlation between PD-L1 expression and treatment efficacy, with higher PD-L1 expression levels associated with greater benefits from immunotherapy in NSCLC patients (58).

Multiple AI techniques have been successfully applied to predict PD-L1 expression levels. Monaco et al. (59)constructed a tri-variate linear discriminant model based on ML algorithms, extracting metabolic parameter features from PET/CT images, achieving a sensitivity of 81% and a specificity of 82% in the test set. DL techniques can also be employed to identify the expression of PD-L1 in lung cancer tissues, with strong objectivity and repeatability of the results, eliminating human errors. Yang et al. combined radiomics with laboratory and clinical data to develop and validate a DL model for the identification of immunotherapy responders and non-responders. This model predicts the response of advanced NSCLC patients to anti-PD-1/PD-L1 drugs with an AUC of 0.80 (60).

AI systems have achieved promising results in predicting PD-L1 status. Clinical indicators and radiomics features play complementary roles in the prediction, providing accurate estimates for determining PD-L1 status (61). Combining various medical imaging and biological data, such as PET/CT, genomic data, and clinical information, through the comprehensive analysis of multimodal data, could lead to the identification of additional potential predictive factors, thereby enhancing the accuracy of the prediction model. These may represent potential directions for further development.

## 5 Prognosis

Compared to traditional prognostic prediction based on clinical features, AI-assisted prognostic prediction is more accurate and efficient (62). Trebeschi et al. (63) trained a neural network to identify morphological changes observed in retrospective chest CT scans of stage IV NSCLC patients during follow-up. They used a classifier to link the learned radiomic features with OS. The results showed significant performance in predicting 1-year OS from the time of image acquisition, with an average AUC of 0.69. The highest AUC occurred within the first 3-5 months of treatment, reaching 0.75. The AUC for predicting sustained clinical benefit (6-month PFS) was 0.67.

Conventional analysis of single-plex chromogenic immunohistochemistry (IHC) focused on quantitative but spatial analysis. AI algorithms can perform quantitative and spatial analysis of immune checkpoint expression and extract prognostic features from IHC images to assist the prediction of immune checkpoint features in survival and relapse. Guo et al. (64)utilized DL to analyze IHC pathology images and constructed a prognostic prediction model. U-net was applied to segment tumor cells and tumor-infiltrating lymphocytes, while ResNet was performed to extract prognostic features from IHC images. The results showed that the model achieved AUCs of 0.9 and 0.85 for predicting OS and relapse-free survival (RFS), respectively.

## 6 Limitations

Although AI has made significant progress in the field of lung cancer, it may still require some time to be fully integrated into clinical practice. Overall, the application of AI in lung cancer still faces certain limitations and challenges:

1. Database: AI models necessitate a substantial amount of data for training, and the quality of this data is crucial for the accuracy and reliability of the predictions. Nonetheless, certain data may suffer from issues such as missing information, biases, and noise, all of which can adversely affect the performance and predictive efficacy of the models. Furthermore, variations in data across different hospitals, including differences in equipment, techniques, and treatment protocols, can also influence the accuracy and reliability of the data. Through collaborative efforts to create standardized datasets and benchmarks, the variability in data quality can be reduced, thereby enhancing the robustness of AI applications.

2. Interpretability: The interpretability of AI algorithms remains a challenge. ML and DL techniques are widely applied in lung cancer research, but these models often function as black-box models, making it difficult to understand their decision-making process and underlying reasons. This makes it challenging for doctors to interpret the output results of the models, posing risks and challenges in practical applications. Fortunately, Interpretability of AI systems is a quickly growing field that has been highlighted by the radiology community as an important area of development, with much potential for the development of safe and intelligible AI technologies (65).

3. Generalization issues: Generalization refers to the ability of a model to learn from a given set of data and apply the learned model to other domains. Unlike natural images, medical images exhibit significant distribution differences when trained DL models are applied to datasets from different vendors. As a result, the generalizability of models becomes a major concern (66).

4. Ethical considerations and privacy: When utilizing patient data for predictions, ensuring data security, privacy, and proper regulation of data usage are essential. AI should be employed as an aid in medical diagnosis and not as a standalone diagnostic tool. Overreliance on AI for diagnosis poses significant safety risks. Furthermore, addressing potential biases in AI models is crucial, as they can lead to unfair treatment outcomes.

5. Legal Issues: The application of AI in healthcare also faces legal and liability challenges. The current ambiguity in legal handling of artificial intelligence will profoundly impact the development of autonomous AI. Advocates of artificial intelligence in radiology and healthcare need to lobby for legislative action to better clarify the liability risks of AI without hindering technological advancement (67).

Additionally, AI models require rigorous prospective validation studies to ensure their efficacy and safety in clinical settings. If not properly managed, AI could potentially exacerbate existing healthcare disparities.

## 7 Conclusion and outlook

AI technology plays a crucial role in various aspects of lung cancer, including diagnosis, classification, and treatment. It aids healthcare professionals in enhancing their work efficiency and accuracy, facilitating precise allocation of medical resources, and offering optimal treatment strategies for patients. This will significantly enhance the level of lung cancer diagnosis and treatment and improve patient survival rates.

In the future, the application of AI in the field of lung cancer is expected to become more extensive and profound. Firstly, with ongoing optimization of algorithms and an increase in training data, AI can assist healthcare professionals in more accurately detecting abnormalities in lung imaging examinations. Secondly, AI can provide personalized treatment recommendations by establishing precise personalized treatment plans based on patients’ genetic information, clinical characteristics, and treatment responses. Real-time monitoring of treatment effectiveness is another potential application. By combining sensor technology and monitoring data, AI can achieve real-time monitoring and adjustment of treatment effectiveness for lung cancer patients. AI-assisted surgery is also possible, where the combination of robotic surgery and virtual reality technology can assist surgeons in performing precise operations, reducing surgical risks and complications. Lastly, AI-assisted clinical decision-making can be achieved. By utilizing AI techniques, intelligent decision support can be provided during the patient’s diagnosis, treatment, and prognosis assessment processes, enabling personalized, accurate, and efficient clinical management.

In conclusion, the advancement of computer technology, along with the accumulation of medical knowledge and relevant datasets, will lead to an increasingly significant role of artificial intelligence in every aspect of lung cancer, ultimately enabling precise screening, diagnosis, and personalized therapy. While AI proves to be a valuable tool, it will not replace doctors but rather complement their expertise. The future of medical development lies in harnessing the synergies between AI and healthcare professionals to deliver more effective, patient-centered care and drive advancements in lung cancer management.
